# Covert audio recording of the clinical encounter to identify learning needs and exemplary performance in a pediatrics residency program

**DOI:** 10.1371/journal.pone.0344904

**Published:** 2026-03-26

**Authors:** Justin Salehi, Alan Schwartz, Saul J. Weiner

**Affiliations:** 1 Rancho Family Medical Group, Temecula, California, United States of America; 2 Departments of Medical Education and Pediatrics, College of Medicine, University of Illinois at Chicago, Chicago, Illinois, United States of America; 3 Departments of Medicine and Pediatrics, College of Medicine, University of Illinois at Chicago, Chicago, Illinois, United States of America; 4 VA Center of Innovation for Complex Chronic Healthcare, Jesse Brown VA Medical Center, Chicago, Illinois, United States of America; Touro University California College of Pharmacy, UNITED STATES OF AMERICA

## Abstract

**Background:**

Physicians-in-training receive feedback based on assessment of their observed clinical skills in both the clinical setting and in simulations. These serve as proxy measures for clinician performance – that is, the application of those skills in routine clinical practice when “no one is watching.” In research employing covert audio recording of internal medicine residents to directly assess clinical performance of one competency, contextualizing care, there is a “skills-to-performance gap,” defined as the difference between what clinicians do when overtly observed compared to when covertly observed. Feedback collected based on covert observation has been shown to improve physician performance in adult medicine practice. This feasibility study tests whether covert assessment with feedback can be operationalized in a pediatric residency program employing a modified protocol adapted for the pediatric setting.

**Methods:**

Parents of patients cared for by consented residents were recruited in a waiting room to carry a concealed audio recorder into their child’s appointment. Following the encounter, the audio recording was coded using the Content Coding for Contextualization of Care (4C) supplemented by expert opinion to identify additional learning opportunities. Findings were shared with participating residents followed by a second round of data collection.

**Results:**

Among 50 contextual red flags identified across 38 pre-feedback and 13 post feedback audio recorded visits, residents probed just six of them. Of these, patients revealed four contextual factors. They also revealed four contextual factors without a physician probe. In response to only two of these eight contextual factors, the resident attempt to formulate a contextualized care plan. Feedback opportunities unrelated to contextualization of care were also noted. There was no significant change in resident performance with feedback, although sample size was small.

**Conclusions:**

Concealed audio recording is a feasible strategy for assessing clinician performance and providing feedback in a pediatric residency program.

## Introduction

Evaluation and feedback during residency are most relevant when based on observed clinical performance in patient care settings. [1,2] They are typically conducted by a supervising attending in the context of ongoing care delivery or via structured feedback utilizing an assessment instrument such as a mini-CEX, among a range of options. [3] In surgical fields, the Society for Improving Medical Professional Learning (SIMPL) mobile application provides a point-of-care platform for real-time structured and narrative feedback after procedures [4].

A limitation of direct observation, however, is the potential for learner reactivity when trainees know they are being observed, sometimes called the Hawthorne Effect. [5,6] This occurs when the act of observing a clinician who is aware they are being observed changes their behavior. Two of the authors have documented this phenomenon across multiple studies focusing on assessing one particular clinical competency, “contextualizing care” [6], employing research methods that allow for the comparison of overt and covert observation of clinician behavior.

Contextualizing care is the process of adapting care to the circumstances and behavior of individual patients (e.g., recognizing when a patient’s poor asthma control is secondary to a knowledge deficit in correct use of their inhaler rather than undertreatment of their condition). [7] Failures to contextualize care are termed “contextual errors” because they result in care plans that are not appropriate given a patient’s circumstances or behavior. [8] Contextualization of care is assessed using an audio coding system called Content Coding for Contextualization of Care (“4C”). [9]

We previously conducted several studies in adult care settings that together demonstrate a gap between overtly and covertly observed care across a range of settings that we have referred to as the “skills-to-performance gap.” [6,10,11] For example, an internal medicine resident trained to contextualize care may ask a standardized patient who has lost control of a chronic condition about barriers to medication adherence but fail to do so when covertly audio-recorded seeing a real patient. The gap can be so large that medical students outperform attending physicians when completing the same assignment overtly vs covertly observed. [10,11] Finally, when we tested a curriculum designed to teach trainees to contextualize care, we saw a positive effect of the intervention compared to an untrained control when assessed using overt observation, but that disappeared when the curriculum was re-tested using covert observation. [11,12]. We characterize the gap as a difference between skill and performance because overt observation captures what a clinician *can* do, whereas covert observation documents what they *do* do in practice. [6]

To assess and provide feedback on performance, we have developed and widely employed in adult medicine a data collection process termed “patient-collected audio for audit & feedback.” [13] It has been successfully embedded for a decade in one large clinical practice that includes internal medicine residents and for three years in several other adult care settings. [14] Overall, we have employed patient-collected audio in over 10,000 adult medicine clinical encounters. [6] We have demonstrated that sharing clinician performance data with participating clinicians leads to improved performance and better health care outcomes for their patients. [13,15]

The feasibility and educational value of patient-collected audio in pediatrics remains unknown. Pediatric encounters differ from adult visits in several important ways: communication often involves both children and their caregivers, ambient room dynamics can be more unpredictable, and cultural norms around privacy and recording may differ for families with minors. It is therefore unclear whether a similar covert recording protocol can be implemented effectively in a pediatric residency clinic, whether such recordings will yield actionable feedback for trainees, and whether important learning opportunities might be identifiable beyond the structured 4C framework traditionally used in adult studies.

This feasibility study therefore examined: (1) whether a patient-collected covert audio program could be implemented in a pediatric residency clinic; (2) whether such recordings would provide actionable feedback for residents based on 4C coding; and (3) whether additional meaningful learning opportunities beyond 4C could be identified through open-ended review by attending physicians.

## Materials and methods

The protocol for assessing the feasibility of a patient-collected audio audit & feedback program in the pediatric residency at the University of Illinois at Chicago (UIC) is based on one first described in a 2013 publication and subsequently on a Department of Veterans Affairs website, with two modifications as noted below. [12,14] This study was approved by the Institutional Review Board of the University of Illinois at Chicago (STUDY2021–1472-MODCR001). Written consent was obtained from all subjects.

One of us (JS) presented the project at several noon resident conferences and invited interested residents to assent to the study. Those who assented were then contacted to complete the consent process. Residents were assured that the audio recordings would not be shared outside the research team, and that any data used for feedback or publications would be de-identified.

The protocol for participating residents was as follows: Parents or other caregivers of their patients were recruited in the waiting room at the time of their clinic visits when research staff or trained IRB approved volunteers were available. This differed from the adult medicine protocol, in which the patients themselves consent. Front desk staff distributed a pamphlet describing the study, and those who expressed interest were approached by one of the authors (JS) or an IRB approved volunteer. Caregivers who consented were encouraged to keep the recording device concealed but reminded that they could reveal or turn it off at any time, per protocol.

After the visit, caregivers returned the recording device, and the audio file was uploaded to a secure server approved for patient-sensitive data. A member of the research team (JS) -- a board-certified pediatrician and clinician educator trained in 4C -- listened to and coded the recording. Before coding for this study, JS was trained by an experienced 4C trainer who validated his coding accuracy. The 4C system tracks four elements of physician-patient interaction: [9] (a) The presence of a “contextual red flag” which is any indicator the patient may be facing a challenge self-managing their care, ranging from missed appointments to comments suggesting confusion about their treatment plan (e.g., “Sorry I couldn’t make the last few appointments.”); (b) a “contextual probe” which is any question by the physician indicating they are trying understand what challenges the patient might be facing (e.g., “I noticed you’ve missed some appointments. Anything going on that we might help you with?”); (c) a “contextual factor” which is any disclosure either in response to a probe or spontaneously that accounts for the contextual red flags (e.g., “Well, unfortunately my car got impounded”); and (d) a “contextualized care plan” which refers to any attempt by the physician to address the contextual factor (e.g., “Sorry to hear that. We can provide vouchers for you to come on public transportation.”).

In addition to the 4C coding, the coder added a supplemental step: documenting any resident behaviors that represented learning opportunities outside of the 4C framework, including exemplary behaviors or areas for improvement. These observations were intended as formative clinician-educator feedback analogous to what supervising attendings routinely provide based on direct observation, rather than as a formal qualitative thematic analysis. This was the second modification to the adult medicine protocol.

Midway through the study one investigator (JS) provided feedback to residents using examples of both effective and missed opportunities to identify and address contextual factors relevant to patient care. The feedback was presented in person, and the slide deck was emailed to all participating residents afterwards.

### Data analysis

Based on the 4C coding of each visit, we counted contextual red flags, contextual probes, contextual factors, and contextualized plans. We compared the likelihood of probing and contextualized planning before and after the feedback using binomial mixed effects regression models with random intercepts for provider and visit to accommodate the hierarchical structure of the data.

We also sorted non-4C examples into three categories of resident-patient or resident-family interactions that offered educational value: (a) *Positive feedback opportunities* defined as those in which a resident navigated a challenging clinical or interpersonal situation in an unusually productive way; (b) *Constructive feedback opportunities* in which a resident apparently missed an opportunity to effectively manage a clinical or interpersonal interaction; and (c) *Error correction opportunities* in which a resident conveyed information to a patient that is factually incorrect or exhibited behavior inappropriate to the clinical situation.

## Results

**Fig 1:**
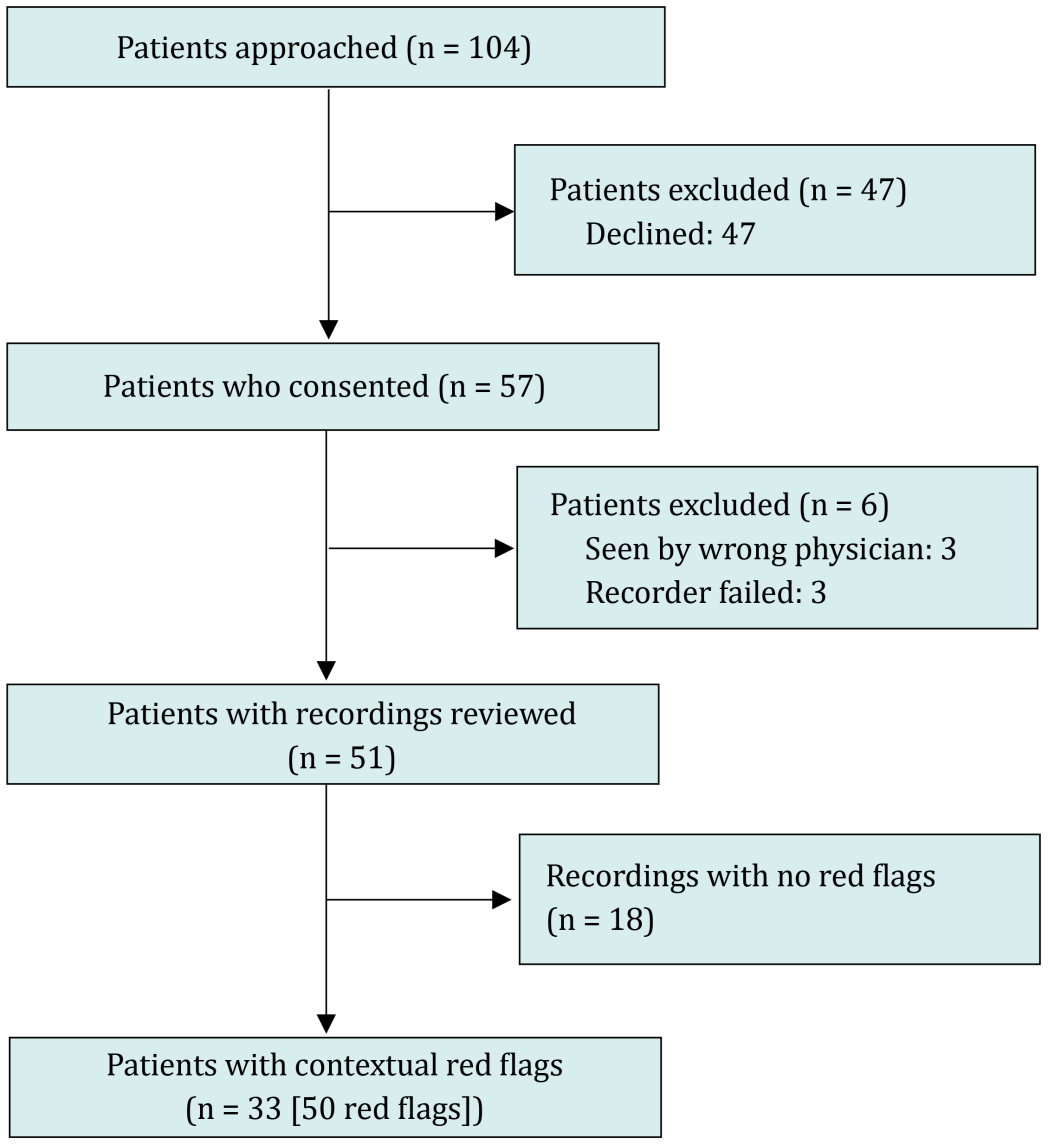
Study Flow Diagram.

From January 17 to June 21, 2024, 17 pediatric and medicine-pediatric residents out of 51 consented to participate. Eight were post graduate year (PGY) 1, four PGY 2 and five PGY 3. Eleven of them were recorded on at least one occasion by 57 caregivers who separately consented to record their visit. Baseline data was collected from January through April (Fig 1).

Feedback was provided to residents at the beginning of May to determine whether performance might change after identifying potential areas for improvement. A total of 51 recordings were analyzed, among which 38 were from the pre-feedback period and 13 post-feedback. All but one recording were made covertly, meaning that it was not revealed to the provider during the encounter.

### Findings from contextualizing care analysis

As shown in Table 1, among 50 contextual red flags, residents probed only six. Of the eight contextual factors identified, four were revealed in response to a resident’s probe and four were disclosed spontaneously. Only two care plans were contextualized. There were no significant differences in the rates of probing or contextualizing care between pre- and post-feedback visits.

**Table 1:** Findings from 4C Analysis.

		Pre-feedback (38 visits)	Post-feedback (13 visits)	P (Post vs. Pre), adjusted for clustering in providers and visits
Red flags	Total	39	11	
	Mean per-visit (SD)	1.03 (0.94)	0.85 (1.07)	
Probes	Total	4	2	0.48
Contextual factors	Revealed in response to a probe	3	1	
	Revealed by parent without a probe	4	0	
Contextualized plans	Total	2	0	1.0

Table 2 shows the eight contextual factors and, for each, indicates whether the initial contextual red flag was probed by the resident or whether the parent revealed the factor without being asked about it. The rightmost column indicates whether the resident addressed the contextual factor in the care plan. For example, after a resident probed missed appointments, a parent disclosed that a recent move made it more difficult to make it for visits, whereas another parent – without probing -- described difficulty managing their child’s tantrums because of work responsibilities at home. In the latter case, the resident involved social services (a contextualized care plan) whereas no plan was developed for the former.

**Table 2:** 4C coding for the eight instances in which a patient revealed a contextual factor either in response to a probe or unsolicited.

Contextual Red Flag	Contextual Probe	Contextual factor	Contextual Care Plan
Patient has missed multiple appointments	“Was anything going on that you didn’t come back after your baby visit but before now?”	Parent stated that they moved, and it’s become difficult to make it for visits	None
Parent declines Rotavirus vaccine after accepting others	“Why did you refuse the Rotavirus and you accepted the others?”	Parent worried about side effects based on information she’d heard	None
Ultrasound ordered 2 months ago for liver lesion but not yet done	“Did they want you to get the ultrasound before going to the GI doctor?”	Patient unaware they should get ultrasound done before seeing specialist	None
Parent not providing routine circumcision care as directed	“What did they tell you to do? To put the cream on every [diaper change]?”	Parent stated, “Yeah I kinda be like scared” about their child’s clinical condition	Resident reviewed how to apply cream and confirmed that parent was comfortable doing it
Family declines Covid vaccine	None	Mom translated for dad that “He’s just worried about how he [their child] keeps getting fevers out of nowhere.”	None
Parent declines flu vaccine	None	Parent states that she isn’t in daycare and so she doesn’t feel like patient needs it.	None
Parent mentions they are having trouble managing child’s tantrums	None	“It’s kind of hard for me [to manage the child] because…he throws a tantrum and I’m on the phone with a client…”	Discusses arranging pediatric social worker to contact parent and arrange for supportive services
Progressive obesity and lack of follow through on recommendations from prior visits.	None	When the resident recommends a nutrition program, parent reveals that it would be difficult for patient to attend because parent is at work on the days that it is offered. She also asserts that she does not have adequate social support stating, “it’s hard.” She notes that patient “can’t participate” in after-school programs since nobody is available to pick child up.	None

For none of the remaining 40 unprobed contextual red flags did a parent spontaneously reveal a contextual factor. These red flags included missed appointments, multiple emergency visits for primary care needs, running out of a daily medication (e.g., a control inhaler) without refilling it, and statements suggesting difficulty attending follow up appointments. In each case, the resident did not probe and the parent didn’t offer an explanation (i.e., a contextual factor).

### Findings from the supplemental analysis

Examples were identified in each of the three categories, as follows.

A positive feedback opportunity:

A resident interacts playfully with a highly active 18-month child who is demanding their attention, while also staying focused on history taking with the parent:

Resident (R): “Did you give him anything?”

Parent: “I gave him Motrin. For now, it’s just a cough though.”

R: “Do you think he’s doing better? Is he eating, drinking…”

Patient: “Come on!” (Demanding to play with pediatrician)

R: “(to patient) “Come on, high five, let’s do it. Good job!” and (to parent) “And how about peeing, pooping?”

Patient: “DAAAA?” (seeking more attention from resident)

R: “(to patient) Yeah, there you go! Good job! (while engaging and playing with patient), and (to parent) “…and then is he going to daycare or anything?”

The above excerpt reflects an unusual capacity to manage both the child and the parent productively in a challenging encounter. The tone remained playful yet focused. This stood in contrast to other recorded encounters in which residents and parents struggled with chaotic exam-room dynamics.

A constructive feedback opportunity:

During a 4 month well child visit, immigrant parents – still acquiring basic English language skills -- expressed persistent level of anxiety about their child’s umbilicus. The resident responded with brief reassurance but repeatedly interrupted the parent without exploring the source of the parents’ concern::

Parent (P): “We thought maybe the bellybutton falls out of place?”

Resident (R): “It seems okay to me. I didn’t notice when I looked at it…those are the big things I would look at: If it’s super red, or if it’s changing color, or becoming super warm to the touch. And it doesn’t seem like it’s doing any of those things.”

P: “But we probably wouldn’t notice if it’s too hot...Is it starting from inside of his guts? How do they put? Rings?”

R: “I wouldn’t worry about anything like that.”

P: “What do you think? Then why?”

R: “I think it’s normal. Like the belly button just has a little bit of…but it’s nothing to worry about.”

P: “But still the bellybutton is like...”

R: [Resident interrupts parent] “The bellybutton looks normal.”

P: “Sometimes the intestines becomes like...”

R: [Resident again interrupts] “No, no it’s not the intestines. There’s nothing wrong with the intestines.”

P: “Could it be like hernia? I don’t know how it’s called but I know a lot of have hernia where the intestines go outside of the muscles...”

R: “No, he’s doing okay. Nothing like that. No need to google things, dad.”

P: “No, I don’t mean...I’ve seen people’s kids with a hernia...”

R: [Resident interrupts parent] “Yeah, he doesn’t have a hernia. For sure he doesn’t have a hernia.”

P: “Okay...Yeah I know but there’s something there. It doesn’t look normal. You’re telling me that it’s not injured, it’s just a little darker?”

R: “I just checked it, it looks normal guys. Don’t worry.”

Residents were also heard using medical terminology that was unfamiliar to parents, leading to confusion. For example, one resident assumed a parent knew the difference between a “general check-up” and a “follow-up appointment,” only to discover that the parent believed the former referred to an acute visit for illness.

An error correction opportunity:

In another example, a resident explained to the parents of an 18-month old that excessive cow’s milk consumption may lead to iron deficiency anemia, but provided incorrect information about the physiological mechanism.

Resident: “He needs to drink less than 24 oz of milk a day…let me tell you why he needs to drink less than that. If you drink too much milk, it can cause teeny-tiny bleeds in your stomach, and if you have that for a long time, you can get anemic from that.”

In children in this age group, cow’s milk’s contribution to iron deficiency is related to its low iron content relative to other competing food sources a child could be consuming, rather than to any harm to the gastrointestinal track. [16] This error is clinically significant because it precludes parents from getting useful information about how to treat or prevent iron deficiency anemia through iron rich age-appropriate food sources.

In a separate exam, the concern was not misinformation but apparent mismanagement. A 30-month-old patient with high-risk asthma presented in acute respiratory distress. Rather than immediately assessing the patient, including checking oxygen saturation, the resident spent six minutes taking a history that included tangential questions (e.g., about siblings or sleep patterns). The child was subsequently admitted for inpatient care, raising concerns that the resident did not recognize the seriousness of the patient’s clinical status or had difficulty prioritizing clinical tasks.

## Discussion

This feasibility study demonstrated the potential for enhancing pediatric resident education through feedback based on analysis of audio recordings collected covertly during clinic visits by patients’ caregivers. Several findings merit emphasis.

Despite its small size, the study yielded useful examples to help pediatric residents better understand the strengths and weaknesses of their approaches to care. Foremost, the 4C analysis revealed frequent missed opportunities for residents to probe contextual red flags, and when they did probe the yield of actionable information was high. Consistent with adult studies employing 4C, this pattern reinforces the importance of probing contextual red flags rather than waiting for patients to share, unprompted, the challenges they are facing self-managing their or their child’s care. [6] In this small data set there were 40 instances in which residents didn’t ask parents about missed appointments, repeated emergency department visits, unfilled prescriptions, or stated barriers to care. It is unlikely that much of these data would have been captured without an audio recording program.

Beyond the 4C analysis, the supplemental data also provided valuable learning opportunities. As noted, these examples expanded the ranges beyond contextualization alone. In the first, a resident skillfully engaged an active child while maintaining a productive clinical conversation with the parent -- a common but challenging scenario in pediatrics. In the second, a resident appeared to struggle to manage a parent’s persistent anxiety over a benign physical finding. Both examples provide rich material for group discussion and reflection to strengthen residents’ relational and communication skills.

The third example, involving a misunderstanding about the mechanism of iron deficiency anemia in a particular clinical setting, demonstrates how audio recording can reveal gaps in knowledge that affect care. Such findings can inform program curricular development; For instance, a discussion about the role of diet in iron deficiency anemia could be added to noon conference topics.

Taken together, these findings suggests that covert observation utilizing patient collected audio may supplement the standard approach of overt observation by attending pediatricians, as the latter is often infrequent, unstandardized, fragmented, and subject to learner reactivity. [17] Furthermore, while some of the performance deficits identified in this study might have been captured through overt observation, the latter is not designed to detect recurring lapses such as frequent missed contextual red flags or failures to address contextual factors. Overt and covert observation are complementary.

This study had limitations. Foremost, the sample size was too small and the feedback too limited to determine whether the intervention could change pediatric residents’ performance, as has been demonstrated in adult settings. Another limitation is that 4C coding was conducted by a single trained coder. Although the senior author verified the coding, additional data may not have been captured. In addition, supplemental observations beyond 4C were generated by a single clinician-educator and were intended as formative educational impressions rather than independently corroborated qualitative findings. An inherent limitation of the intervention is the need for trained 4C coders and clinician-educators to review recordings. Although training materials exist and coding can be learned relatively quickly, the process remains resource-intensive. Ongoing work to automate 4C coding through artificial intelligence may help reduce this burden. [18]

Finally, while the evidence for participant reactivity in the assessment of the competency of contextualizing care is strong, as described above, we cannot generalize that other competencies (e.g., suturing a wound or performing a relevant physical exam) are as prone to a skills-to-performance gap. There is remarkably little research comparing overt to covert observation in health care, although it has been shown to matter when assessing handwashing. [18]

Further research is needed to determine whether ongoing, individualized feedback based on covert audio review can change pediatric resident behavior. Larger studies will also be important to assess stakeholder comfort with audio recording in pediatric settings, which was not systematically evaluated here. In adult settings, patient surveys and provider focus groups have found minimal concerns, especially with repeated exposure. [19] Similar research in pediatric environments is warranted.

## Conclusion

Despite inherent logistical and patient privacy challenges, audio recordings collected by patients to provide clinician feedback based on 4C analysis have been employed at scale in adult care setting. This study demonstrates how a modified protocol was successfully introduced in a pediatrics residency clinic, with the 4C analysis supplemented by additional observations from a pediatric clinician-educator. The analysis revealed a range of trainee behaviors that would likely have gone unnoticed through traditional observation alone. From the 4C analysis, the most notable findings were frequent missed opportunities to probe contextual red flags that parents were struggling with life circumstance or behaviors complicating their children’s care and, when evident, to address them. Additionally, a pediatrician clinician-educator extracted, for teaching purposes, both exemplary resident behaviors as well as missed opportunities in resident–parent or resident–child interactions and errors in practice.
